# The modulation effect of cognition on mentalization in late-life depression: a study of gaze perception—a potential screening tool for high-risk group of late-life depression

**DOI:** 10.3389/fpsyt.2024.1199119

**Published:** 2024-05-01

**Authors:** Sze Ting Joanna Ngan, Calvin Pak Wing Cheng, Wai Chi Chan, Kam Hung Harry Tsui, Sau Man Corine Chan, Kit Wa Sherry Chan

**Affiliations:** ^1^ Department of Psychiatry, University of Hong Kong, Hong Kong, Hong Kong SAR, China; ^2^ Department of Psychiatry, Queen Mary Hospital Hong Kong, Hong Kong, Hong Kong SAR, China; ^3^ Department of Public Health, University of Hong Kong, Hong Kong, Hong Kong SAR, China

**Keywords:** late-life depression, gaze perception, social cognition, mentalisation, early detection

## Abstract

**Introduction:**

Impairment in mentalization is implicated in the development and maintenance of depression. Major depressive disorders showed significant impairment in social cognition and such impairment appears to be positively associated with the severity of depression. Self-referential gaze perception (SRGP), a measurement of mentalization, was predominantly measured in patients with psychosis but rarely examined in late-life depression (LLD).

**Methods:**

To assess the effect of cognition on the interpretation bias of mentalization, 29 LLD patients and 29 healthy controls were asked to judge if various gaze directions were directed to self in SRGP.

**Results:**

Patients with better cognition showed less unambiguous-SRGP bias than those with worse cognitive scores; this difference was not found in healthy controls. Global cognition and executive function contributed to the SRGP rate in patients.

**Conclusion:**

The current study is the first study to explore the relationship between cognition and SRGP in the LLD population. Our study findings suggested that the cognitive function of LLD patients may contribute to the modulation of interpretation bias, which in turn underlie the role of SRGP bias. Greater SRGP bias in patients may reflect social cognition deterioration, impairing the social interaction and functioning of LLD patients. This highlights the need for early intervention and cognitive decline identification to facilitate better prognosis and treatment effectiveness; thus, further studies could navigate the potential of SRGP task as a screening tool for high-risk group of LLD likely to develop dementia.

## 1 Introduction

The high prevalence and poor treatment outcomes of geriatric depression have become a pressing health care issue in Hong Kong. It is estimated that one in six people worldwide will be aged at least 60 years old by 2030 (1). A local study found that around 10% of community dwellers (2) aged 60 years or above suffered from clinically significant depression, referred to as late-life depression (LLD) (3). Compared with depression in adults, LLD is implicated with greater impairment in executive functions and psychomotor retardation (3). Furthermore, LLD is also associated with poor long-term prognosis and a higher relapse rate (4). Most importantly, LLD is related to an increased risk of cognitive impairment and dementia (5). Hence, it is important to examine geriatric depression as a separate and unique entity. Also, it is of importance to identify people at risk of LLD of further cognitive impairment.

Mentalization, as a form of social cognition, infers the ability to identify, perceive, and interpret socially relevant information (6). Mentalization impairment in depression is partly contributed by the inability to interpret emotional stimuli and mental states accurately (7). The negative interpretation bias, which is the tendency to infer ambiguous social information as negative valence, has been identified as the core feature of depression for decades (8). Recent theory suggests that interpretation bias plays an important role in the onset and maintenance of depression (9, 10).

Self-referential gaze perception (SRGP) was predominantly measured in patients with psychosis but rarely in the depression cohort. Tendence of perceiving averted gaze as self-referential was more prominent in bipolar disorder and schizophrenia patients than healthy adult controls (11, 12). For depression patients, biased self-referential processing was commonly measured with the interpretation of cognitive information related to self-schemas. Depressogenic self-schemas measured by self-referential encoding task were more prevalent in patients with depression than controls in children, adolescents, and adults (13–15). While self-referential bias was implicated in depression consistently across different age groups, no studies, to our knowledge, explored self-referential processing among old-age adults with depression. Existing literature indicated healthy older adults had poorer social cognitive function than the younger adults (16). Other suggested that the impairments vary between subsets of social cognition where attribution process in older adults declined but emotional perception remained intact (17–20).

Judgment of self-referential gaze intention with gaze perception task is a relatively simple assessment of mentalization, which is an important component in social cognition. However, limited studies to date explored mentalization of LLD and to our knowledge, no studies examined eye gaze interpretation among LLD patients. Therefore, the current study aimed to examine the SRGP of patients with LLD compared with healthy controls (HCs). We hypothesized that patients would have greater SRGP bias than HCs. The secondary aim of the study was to explore the relationships between symptomology, global cognition, and gaze perception. We hypothesized that both depressive symptoms and global cognition would be related to SRGP bias. The findings of this study will help to provide further understanding in the interpretation bias in LLD, hence, develop a screening tool for detecting high risk LLD.

## 2 Materials and methods

### 2.1 Sample

Fifty-eight Chinese older adults aged 60 years or above were recruited, with 29 in patient group and 29 age- and gender-matched health controls. Patients with primary diagnosis of major depressive disorder (MDD) screened by psychiatrists from outpatient psychiatric clinics of the public hospital in Hong Kong with structured Clinical Interview for the Fifth Edition of the Diagnostic and Statistical Manual of Mental Disorders (DSM-5), and no change in dosage of antidepressant for at least 2 weeks was included. Patients with a DSM-5 diagnosis other than MDD [e.g., major neurocognitive disorder of any types, including Alzheimer’s disease (AD)], a score of Hong Kong Chinese version of the Montreal Cognitive Assessment (HK-MoCA) (21) below second percentile relative to the subject’s age and education level, with comorbid alcohol or substance dependence or any concomitant unstable major medical or neurological conditions will be excluded. HCs with the same exclusion criteria, in addition to no personal or family history of mental illnesses, were recruited from the community. Face-to-face interviews were conducted to screen for normal or corrected-to-normal vision and obtain written informed consent. This current study was approved by the institutional review board of the University of Hong Kong and Hong Kong West Cluster of Hospital of Authority (IRB: UW 22-712).

### 2.2 Assessment

Demographics including age, gender, and level of education was collected. Depressive symptoms were assessed with the Hamilton depression rating scale (HAM-D-17 (22). Information regarding symptomology including medical comorbidity and independent functioning ability were assessed by the Cumulative Illness Rating Scale (CIRS) and the Hong Kong Chinese version of the Lawton Instrumental Activities of Daily Living (IADL), respectively. While global cognition was measured by Mini-Mental State Examination (MMSE), working memory and attention were examined by the forward and backward digit span, and executive function was assessed by the Stroop color and word test, Trail Making Test (TMT) and verbal fluency test (VFT).

### 2.3 Experimental task

A gaze perception task developed in the previous study (12) was used. In short, the task consists of six blocks, and each block contains 30 trials of randomly selected facial stimuli of one model with pre-determined distribution of gaze directions and was programmed using E-prime Professional 2.0. There were six Chinese models (three men and three women) facing straight to camera with 13 different gaze directions (0°, 5°, 10°, 15°, 20°, 25°, and 30° to the left and to the right, respectively). Participants were instructed to press the right key “yes” or the left key “no” to the question in evaluation for self-referential gaze: “Do you feel as if the person in the picture is looking at you?” Each trial began with 200 ms presentation of the face, followed by a fixation cross remained on the screen for 1800 ms and ended with a blank page for 500 ms (23). Accuracy and response time were collected automatically by the program within the 2500 ms time frame in each trial. Missing responses in the 2500 ms were coded as nil response. All participants would begin with a short practice run of the task using new stimuli before the start of the experiment. The experiment lasted for approximately 20–25 min.

### 2.4 Data analysis

Ambiguous-SRGP rate and unambiguous-SRGP rate were calculated for the gaze perception task as suggested in the previous study (12). Ambiguous-SRGP rate was indicated by the average rate of reporting the gaze as self-directing (SRGP) with trials of averted gaze direction of 10° and 15°, whereas the unambiguous-SRGP rate was the average rate of reporting the gaze as self-directing (SRGP) with trials of averted gaze direction of 20°, 25°, and 30°. Demographic, clinical, and cognitive measures were compared between patient and control groups using paired samples ***t***-tests for the parametric variables and Mann–Whitney ***U*** tests for the non-parametric variables. Group differences in ambiguous-SRGP, unambiguous-SRGP, depressive symptomology, and cognitive function were analyzed using generalized linear model, controlled for age, gender, and education years. Considering the non-parametric nature of the variables, Spearman’s correlation was performed to examine relationship between gaze perception, cognitive functioning, and symptomatology. ***P***-values were adjusted (***p*** = .001) for multiple testing using with Benjamini–Hochberg false discovery rate correction (FDR = 0.1). Linear regression was used to explore the contribution of cognitive functions on SRGP. All tests would be two-tailed with the significance level set at ***p*** < 0.05. Analyses were performed with SPSS for Windows, version 27.0.

## 3 Results

There was no significant difference in age, gender, and education years between group (**Table 1**). Also, no significant difference was found in cognitive functions, but among symptomology, significant group difference was found in the HAM-D scores.

**Table 1:** Group difference in demographics, cognitive function, and symptomology.

	Patients (*N* = 29)	Healthy controls (*N* = 29)	*U*	*P*
Demographics				
Age, years	70.24 (5.12)	69.48 (5.48)	.578	0.588
Male, *n* (%)	11 (37.9%)	11 (37.9%)	.000	1.000
Education, years	7.93 (4.43)	8.62 (4.63)	.102	0.565
Symptomatology				
HAM-D	8.14 (4.76)	4.41 (5.06)	212.50	0.001
CIRs	5.27 (3.51)	4.38 (3.26)	369.50	0.423
ASEC	48.77 (6.73)	51.14 (6.78)	249.00	0.182
SHAPS	0.95 (2.75)	0.31 (.71)	288.00	0.280
IADL	25.54 (2.06)	26.14 (1.61)	330.50	0.132
Cognitive functions				
MMSE	26.81 (2.26)	27.11 (2.77)	299.00	0.660
Stroop	23.99 (11.88)	20.17 (10.02)	348.00	0.355
TMT	45.73 (33.42)	37.54 (29.65)	317.00	0.155
Digit span forward	8.18 (1.09)	8.51 (0.75)	341.50	0.253
Digit span backward	3.86 (1.78)	4.78 (1.85)	308.00	0.111
VFT60	41.86 (8.76)	43.04 (7.08)	405.50	0.815
Self-referential gaze perception				
Unambiguous	0.14 (0.16)	0.0885 (0.088)	349.50	0.138
MMSE < 27	.26 (.22)	.11 (.11)		
MMSE ≥ 27	.073(.11)	.074(.077)		
Ambiguous	0.30 (0.21)	0.29 (0.21)	404.00	0.144
MMSE < 27	.44 (.066)	.35 (.066)		
MMSE ≥ 27	.20(.096)	.45(.049)		

All values are presented in the form of mean (SD). HAM-D, Hamilton Depression Rating Scale; CIRs, Cumulative Illness Rating Scale; AES-C, Apathy Evaluation scale; SHAPs, Snaith-Hamilton Pleasure Scale; IADL, Instrumental Activities of Daily Living Scale; MMSE, Mini-Mental State Examination; TMT, Trail Making Test; VFT 60, verbal fluency test 60th percentile.

### 2.1 Group difference in SRGP

No significant group difference was identified for ambiguous-SRGP (*U* = 404.00, *p* = 0.144) or unambiguous-SRGP rate (*U* = 349.50, *p* = 0.138). A two-way analysis of variance (ANOVA) was conducted to explore the interaction effect of subject group and cognitive function (**Table 2**). Global cognition was compared using median split of MMSE, that is 27 as cutoff. There was statistical significant interaction effect, [*F*(1, 47) = 4.719, *p* =.035], main effects of subject group, *F*(1,47) = 4.631, *p* = .037, and MMSE severity, *F*(1, 47) = 10.010, *p* =.003, on unambiguous SRGP rate. Pairwise comparison revealed higher MMSE scores in patients had a significantly lower unambiguous SRGP (*M* = .074, *SD* = .11) rate than those with lower MMSE scores (*M* = .26, *SD* = .22). No significant interaction effect was found in the ambiguous SRGP task performance (*p* = .252).

**Table 2:** ANOVA on differentiating group difference in cognitive function on SRGP.

	Unambiguous-SRGP	Ambiguous-SRGP
Main group effect (MMSE < 27 vs. MMSE ≥ 27)	*F*(1,47) = 10.010, *p* = .003	*F*(1,47) = 8.657, *p* = .005
Main group effect (patients vs. control)	*F*(1,47) = 4.631, *p* = .037	*F*(1,47) = .109, *p* = .743
Interaction effect	*F*(1,47) = 4.719, *p* = .035	*F*(1,47) = 1.346, *p* = .252

MMSE, Mini-Mental State Examination.

### 2.2 Relationship between demographic, clinical and cognitive function, and SRGP

Relationships between clinical and cognitive function, and SRGP were explored, when age, gender, and year of education were controlled for (**Table 3**). The results of linear regressions indicated that only in patient group alone, MMSE (β = −.060, *p* = .002), CIRS (β = .026, *p* = .018), and digit span forward (β = −.070, *p* = .020) contributed to ambiguous SRGP rate. While CIRs contributed to the unambiguous SRGP in both patients (β = .020, *p* = .021) and in HCs (β = .009, *p* = .039), only MMSE (β = −.045, *p* = .002) and VFT (β = −.008, *p* = .046) contributed to the unambiguous SRGP in patient group. And, digit span forward (β = −.031, *p* = .046) contributed to the unambiguous SRGP only on control group.

**Table 3:** Relationships between SRGP responses, clinical and cognitive information.

	Ambiguous-SRGP				Unambiguous-SRGP			
	All		Patient	Control	All		Patient	Control
	β	R square	R square		B	R square	R square	
Symptomatology								
HAM-D	−.002*	.148	.094	.019	.002*	.152	.187	.181
CIRs	.016*	.203	.326*	.140	.015***	.271	.318*	.280*
ASEC	−.001	.125	.144	.131	−.002*	.155	.215	.241
SHAPs	−.001	.136	.172	.132	.006*	.163	.234	.180
IADL	.014*	.164	.248	.125	.006*	.153	.244	.210
Cognitive functions								
MMSE	−.030*	.253	.521*	.165	−.018*	.257	.521*	.246
Stroop	.003*	.165	.219	.132	.004*	.228	.268	.255
TMT	.001*	.184	.217	.168	.001*	.196	.201	.274
Digit span forward	−.066**	.250	.321*	.205	−.038*	.230	.247	.279*
Digit span backward	−.012*	.168	.356	.150	−.013*	.181	.219	.221
VFT60	−.001*	.147	.185	.125	−.003*	.176	.270*	.179

P-values are adjusted with Benjamini–Hochberg false discovery rate correction procedure for multiple testing (FDR = 0.1). Age, gender, and education years were controlled. HAM-D, Hamilton Depression Rating Scale; CIRs, Cumulative Illness Rating Scale; AES-C, Apathy Evaluation scale; SHAPs, Snaith-Hamilton Pleasure Scale; IADL, Instrumental Activities of Daily Living Scale; MMSE, Mini-Mental State Examination; TMT Trail Making Test; VFT 60, verbal fluency test 60th percentile.

*p < 0.05.

**p < 0.01.

***P < 0.001.

## 3 Discussion

To the best knowledge of the authors, this is the first study to explore the relationship between cognition and SRGP in the LLD population. This study explored the difference in SRGP between LLD patients and HCs. There was a trend for group difference in SRGP bias but has not reached significant level. However, when cognitive abilities were taken into account, patients with better MMSE scores showed less unambiguous-SRGP bias than those with lower MMSE scores, this difference was not found in HCs. Consistent with our hypothesis, global cognition and executive function were associated with SRGP rate. Medical comorbidity was also positively correlated to SRGP rate. Cognitive measurements on global cognition and working memory, as well as medical comorbidity significantly explained the ambiguous-SRGP rate only in patients. Meanwhile, unambiguous-SRGP bias was contributed by the global cognition and verbal functioning in patients, whereas in control group, working memory performance differentiated perception bias.

The null group difference in both ambiguous-SRGP and unambiguous-SRGP may be explained by the limited sample size of this study. In addition, most of the LLD subjects in the current study fall only into mild depression category. As mentioned, MDDs showed significant impairment in social cognition and such impairment appears to be positively associated with the severity of depression (7). Therefore, we suggest a significant group difference may be present when a larger sample size with more severe depression subjects is included for comparison.

Despite no significant difference in SRGP between LLD and HC group, we found that LLD with more cognitive impairment showed a significant SRGP bias compared with those with less cognitive impairment, particularly in the unambiguous stimuli. A possible explanation is that the more difficult condition (i.e., ambiguous SRGP) requires more cognitive resources; thus, patient or HC performed levelly inferior than unambiguous SRGP. In other words, the inherent cognitive bias tendency in depressive disorder only prevailed in the easier condition (i.e., unambiguous SRGP) that requires less cognitive resources, highlighting the influence of possible cognitive bias in LLD but not HC.

LLD subjects with more impaired cognitive function was related to greater SRGP bias in both ambiguous and unambiguous gaze direction, similar to schizophrenia patients (12). In patients with LLD, greater working memory was related to less ambiguous-SRGP bias. Such pattern was not found in HCs. On the other hand, in unambiguous gaze perception condition, global cognition and executive functioning explained unambiguous-SRGP rate in patients. In HC, working memory ability served as the only cognitive indicator to explain unambiguous-SRGP rate. In other words, consistent with our hypothesis, the presence of SRGP bias in LLD patients vary according to the degree of cognitive impairment. Based on this finding, we postulated that the cognitive function in LLD patients plays an unique role in explaining the SRGP bias. The relatively intact cognitive function may help LLD patients to filter the SRGP bias. Regarding only those LLD subjects with significant cognitive impairment displayed SRGP bias, this may be explained by losing the “error detection” function, which believed to be associated with executive function impairment (24). Specifically, our findings revealed better working memory and executive functions in patient had less SRGP bias which was in supportive of our postulation. This adds new evidence into understanding social cognition in depression in older adults.

Existing research revealed that symptomatic psychosis patients displayed higher ambiguous SRGP and were negatively associated with cognitive functions in the psychosis population (12, 25). The findings are consistent with our results. Since cognitive impairment is very common in LLD, we expected that impairment would extend to eye gaze interpretation. In other words, the high SRGP bias in gaze perception task may indicate the presence of a significant cognitive impairment in addition to self-referential bias in LLD, by further replicating and expanding upon the current study, the SRGP task could serve as a potential screening tool to identify the high-risk group likely to develop dementia.

The limitation of this study is the sample size was small. Group difference in SRGP bias was not detected which might be contributed by the limited severity of depression subjects for comparison. In addition, depressive symptoms closely related to gaze perception including thought delusion or reference ideation were not assessed in this study. Therefore, future studies can further explore the SRGP bias in depression patients in various directions. First, future studies can include subjects with from mild to severe depression to further understand the role of SRGP in depression of different severity. Second, the inclusion of assessing thought delusion and thought insertion symptoms in depression patients can help to elucidate if SRGP bias is sensitive towards the particular symptoms that is present in both psychosis patients and depression patients, or it is a unique trait factor of LLD. Our study focused on the role of executive function and SRGP in which other cognitive domains such as memory or visual spatial functioning can be additional measures in future studies to help elucidate the predictive value and differentiation of different types of dementia, including AD, vascular dementia or frontotemporal dementia.

## 4 Conclusions

The present study examined the difference in SRGP bias between LLD patients and HCs. Results revealed that cognitive functioning modulated the interpretation bias in LLD patients, that SRGP is likely to be a trait phenomenon. The greater SRGP bias in patients may reflect social cognition deterioration, impairing the social interaction and functioning of LLD patients. Early intervening and identifying cognitive decline facilitate better prognosis and treatment effectiveness; hence, further studies on gaze perception task could explore the potential of the SRGP task as a screening tool for high-risk group of LLD likely to develop dementia. Future studies could explore the difference in SRGP bias in LLD patients with a bigger sample and greater severity to elucidate the differential effect of cognition to SRGP bias in LLD population.

## Data availability statement

The original contributions presented in the study are included in the article/supplementary material. Further inquiries can be directed to the corresponding author.

## Ethics statement

This current study was approved by the Institutional Review Board of the University of Hong Kong and Hong Kong West Cluster of Hospital of Authority (IRB: UW 22-712). The studies were conducted in accordance with the local legislation and institutional requirements. The participants provided their written informed consent to participate in this study.

## Author contributions

JN drafted and prepared the manuscript. JN, CC, and SC contributed to the interpretation of data. JN and HT contributed to the acquisition and analysis of data. CC, WCC, and SC contributed to the conception and design of the study. All authors contributed to the article and approved the submitted version.
